# Direction-specific interaction forces underlying zinc oxide crystal growth by oriented attachment

**DOI:** 10.1038/s41467-017-00844-6

**Published:** 2017-10-10

**Authors:** X. Zhang, Z. Shen, J. Liu, S. N. Kerisit, M. E. Bowden, M. L. Sushko, J. J. De Yoreo, K. M. Rosso

**Affiliations:** 1 Physical and Computational Sciences Directorate, Pacific Northwest National Laboratory, Richland, 99352 WA USA; 2 Environmental Molecular Sciences Laboratory, Pacific Northwest National Laboratory, Richland, 99352 WA USA

## Abstract

Crystallization by particle attachment is impacting our understanding of natural mineralization processes and holds promise for novel materials design. When particles assemble in crystallographic alignment, expulsion of the intervening solvent and particle coalescence are enabled by near-perfect co-alignment via interparticle forces that remain poorly quantified. Here we report measurement and simulation of these nanoscale aligning forces for the ZnO(0001)-ZnO(000$\bar 1$) system in aqueous solution. Dynamic force spectroscopy using nanoengineered single crystal probes reveals an attractive force with 60^o^ rotational periodicity. Calculated distance and orientation-dependent potentials of mean force show several attractive free energy wells distinguished by numbers of intervening water layers, which reach a minimum when aligned. The calculated activation energy to separate the attractively bound solvated interfaces perfectly reproduces the measured 60^o^ periodicity, revealing the key role of intervening water structuring as a basis to generate the interparticle torque that completes alignment and enables coalescence.

## Abstract (web summary)

Crystal growth is a fundamental process, important in a wide range of fields, but the interparticle forces responsible for molecule alignment are not well understood. Here, the authors measure the alignment forces in ZnO using dynamic force spectroscopy, highlighting the role of intervening water molecules.

## Introduction

Crystallization by particle attachment (CPA) in solution is a widespread phenomenon in geochemical, biomineral, and synthetic material systems^1–4^. Unlike classical crystal growth via ion addition, CPA is facilitated by aggregation of particles, typically nanosized, that coalesce, recrystallize, or assemble into larger structures^1, 4–6^. A complete picture of CPA, of course, also includes classical monomer-by-monomer dissolution, precipitation, and ripening during the particle motion, collision, and aggregation^1^. Oriented attachment (also known as “oriented aggregation”) is a special case in which aggregating nanocrystals self-assemble into extended lattices through preferential attachment on specific crystal faces. Since its discovery in natural systems^2, 3^ there has been growing interest in exploiting oriented attachment to construct hierarchically structured crystalline materials. Examples include metals such as Au^7^ and Ag^8^, alloys such as ZnTe^9^, Pt-Ni, Pt-Cu, and Pt-Fe^10, 11^, metal oxides such as ZnO^12, 13^, TiO_2_^3^, MnO^14^, and α-Fe_2_O_3_^15^, metal sulfides such as PbS^16^, ZnS^17^, Ag_2_S^18^, and CdS^18, 19^, and metal selenides such as PbSe^20^, CdSe^19^, NiSe_2_^21^. Furthermore, surfactants have been widely used in experiments to facilitate oriented particle assembly via preferential adsorption on crystal surfaces. The proposed roles of surfactants in aligning nanoparticles and promoting oriented attachment include surfactant interaction-related driving forces for self-assembly or mesocrystal formation^22–24^, stabilization of kinetically metastable intermediate nanoparticles, and control of coalescence crystal face or the overall shape of the mesocrystal^20, 24^.

Face selectivity that enables oriented attachment conceptually arises from interparticle forces that are both sensitive to mutual crystallographic orientation and strong enough to rotate approaching particles into lattice alignment^1, 3^. However, so far these forces have been more often inferred than measured. For example, recent developments in liquid cell transmission electron microscopy (TEM) imaging technologies have provided opportunities to investigate crystal growth by oriented attachment in situ in real time^11, 25^. Such a study of the iron oxyhydroxide ferrihydrite in aqueous solution showed that, during particle encounters, a nanoscale solvent-separated state was maintained in which nominally random mutual rotational motions were overtaken by stronger directional forces when close to crystallographic alignment, enabling successful oriented attachment and coalescence^25^. For dipolar nanoparticles such as Pt_3_Fe, a strongly anisotropic assembly pattern was observed in which chains were formed by end-to-end attachment conceptually consistent with interparticle dipole alignment^11^. Clearly, assembly is governed by forces capable of creating interparticle torques strong enough to enable alignment while particles are still solvent separated, but to date these forces remain poorly quantified.

It is well-known that the interfacial forces between two flat solid surfaces in solution strongly depend on the atomic structures of the surfaces and the intervening solvent molecules, especially at short range. For example, the interaction between two macroscopic mica basal surfaces in aqueous solution measured using the surface force apparatus (SFA) showed oscillatory repulsive hydration forces consistent with structured intervening water layers, and adhesive force maxima at twist angles of 0° ± 60°, ± 120°, and 180°, consistent with azimuthal alignment of the mica-mica lattices^26^. However, extending these kinds of studies with control of mutual crystallographic orientation and solution conditions into the nanoscale domain of oriented attachment systems is experimentally challenging. One study reported atomic force microscopy(AFM)-based force measurements between a micron-sized oriented gypsum single crystal and opposing substrate^27^. But to understand the forces enabling oriented attachment, measurements of the direction-specific interaction forces on nanocrystals in situ is an essential step. The recently developed in situ environmental TEM-AFM technique also allows measurements of direction-specific interaction forces between nanocrystals but is limited to high-vacuum or low-water vapor conditions^28^.

Here we report AFM-based measurements of the forces on an oriented face-specific single nanocrystal probe as it interacts with its symmetrically related counterpart crystal face in aqueous solution using dynamic force spectroscopy (DFS) (Supplementary Fig. 1). By controlling the azimuthal alignment, the measurements test the dependence of the interaction on crystal symmetry, a fundamental prerequisite of oriented attachment. The novelty of the experimental aspect of our study is precision control of the tip crystallographic face expression, area, and orientation at the nanoscale. Also, by measuring the jump-from-contact force as a function of tip retraction rate, the rupture force at equilibrium and the corresponding adhesion-free energy as a function of azimuthal alignment is directly obtained^29^. We compare the measured orientation dependence of the adhesion-free energy to predictions of the potential of mean force (PMF) from large-scale molecular dynamics (MD) simulations of the solvated interparticle gap. From the combined results we demonstrate that the sensitivity to crystallographic alignment between nanocrystals at close range arises, at least in part, from the self-organization of intervening water molecules in response to the structures and alignment of the opposing surfaces. Furthermore, we show that alignment creates an attractive energy minimum with fewer intervening water layers between the surfaces, enabling closer approach. The findings show that the solvent structure in the interparticle region creates an energy barrier that helps maintain the solvent separated state.

ZnO is an important material in semiconductor and micro-electric industries^30–32^. It has been used to fabricate nanogenerators^33^, solar cells^34, 35^, LEDs^36^, and photo sensors^37^, and as a photocatalyst to degrade organic pollutants^38^. Both theoretical calculations and experimental data have indicated the ZnO nanocrystals oriented aggregate through the (001)/(002) surface to form the rod-like single crystals^12, 13, 39^. Thus our study focuses on Zn-terminated ZnO(0001) interaction with its partner O-terminated ZnO(000$\bar 1$) face as a model oriented attachment system. ZnO has a hexagonal wurtzite-type structure (P6_3_mc), which is composed of alternating planes of tetrahedrally coordinated O^2−^ and Zn^2+^ ions, stacked along the *c*-axis in an ABAB pattern (hexagonal close packing) (Supplementary Fig. 2)^30^. This stacking of oppositely charged ions produces a net dipole moment normal to the basal (0001) plane^31^. An opposing pair of Tasker type III polar bulk truncations is thus obtained, which reconstruct to form stable (0001) and (000$\bar 1$) terminations.

## Results

### Force measurement method

**Fig. 1:**
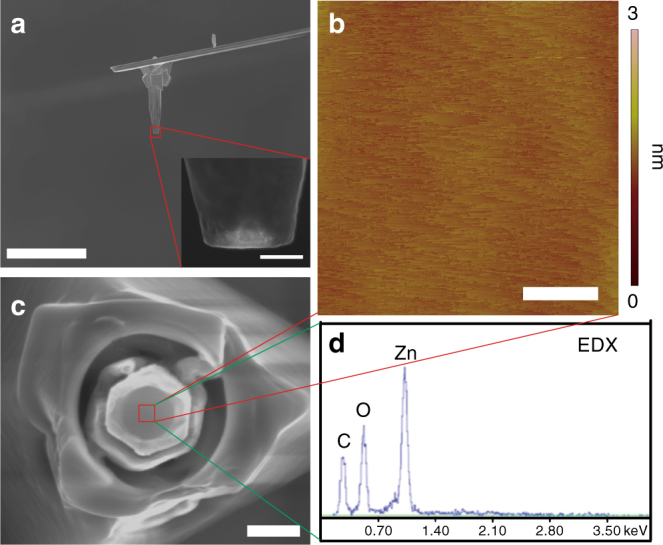
SEM and AFM images of a ZnO(0001) AFM tip. **a** *Side* view (*scale bar* 10 µm), the inset (*scale bar* 100 nm) is a high magnification image to show surface flatness; **b** *Top* view (*scale bar* 500 nm); **c** an AFM image of the ZnO tip surface, collected by reverse imaging using a whisker substrate (*scale bar* 25 nm); and **d** an EDX spectrum of the ZnO tip surface

For our force measurements, a (000$\bar 1$) terminated bulk ZnO crystal was used as a substrate, and (0001) terminated probes were nanoengineered onto AFM cantilevers (Methods section, Supplementary Note 1, Supplementary Figs. 3, 4 and 5). The resulting ZnO(0001) AFM tips (Fig. 1 **)** showed ~ 0.5 nm roughness arising from stepped atomically flat terraces as determined by high resolution reverse AFM imaging, and exposed clean stoichiometric surfaces as determined by cross-sectional high resolution scanning electron microscope (SEM) and energy-dispersive X-ray spectroscopy (EDX). Measurements were performed in flowing 0.2 mM Zn(NO_3_)_2_ solution (Methods section, Supplementary Note 2, and Supplementary Fig. 6). Given the hexagonal symmetry of ZnO along its *c* axis, the azimuthal alignment was varied from 0° to 120° during the force measurements.

### Measured ZnO(0001)-ZnO(000$\bf \bar 1$) interaction force

**Fig. 2:**
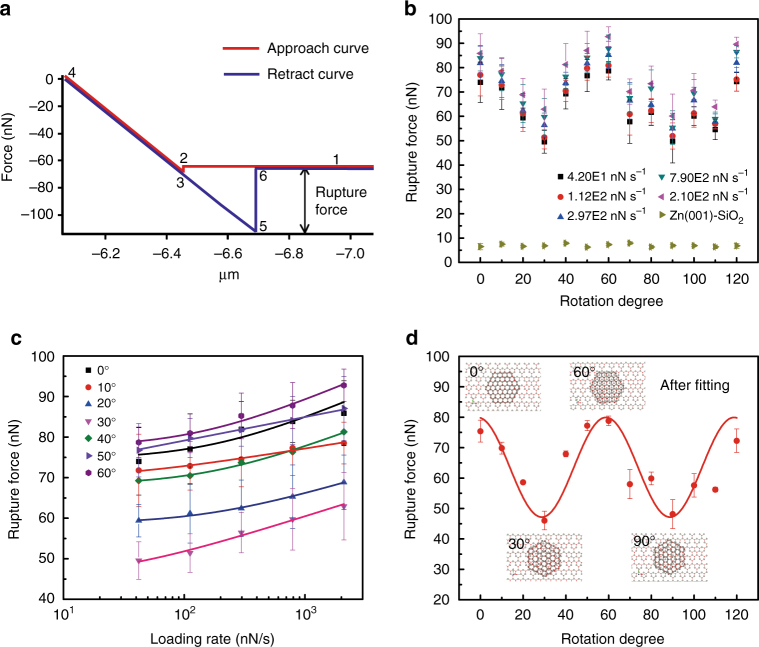
Face-specific interaction force measurements. **a** Representative force curves for ZnO(0001)-ZnO(000$\bar 1$) interaction in saturated aqueous solution. Point 1–2 presents the approach process until the jump-to-contact happened (point 2–3); then further push the tip to contact the substrate from point 3–4; after a short time to stabilize the interaction, tip retraction was performed (point 4–5) until a jump-from-contact happened (point 5–6). **b** Direction-specific interaction forces at different azimuthal orientation for ZnO(0001)-ZnO(000$\bar 1$) and ZnO(0001)-SiO_2_/Si and different loading rates for ZnO(0001)–ZnO(000$\bar 1$). **c** Data fitting for the different loading rates at each azimuthal orientation from 0° to 60° to obtain the equilibrium rupture force. *Error bars* in **b** and **c** represent the s.e. (*n* = 25). **d** Rupture forces at different azimuthal orientation after fitting using the multiple bond model developed by Friddle et al.^29^; *Error bars* derived from the fitting of the multiple bond model. Inset illustrates corresponding structure relationships between the tip and substrate crystals at selected azimuthal orientations (0°, 30°, 60°, and 90°)

DFS was used to investigate ZnO(0001)-ZnO(000$\bar 1$) interaction as a function of azimuthal orientation in saturated aqueous solution^29^. Figure 2a shows a typical force versus distance curve, and the force measurement process is shown in Supplementary Movie 1. Point 1 to 2 presents the approach process, in which the ZnO(0001) AFM tip was pushed to contact the ZnO(000$\bar 1$) substrate via a fixed approaching rate. During tip approach a jump-to-contact event was always evident (point 2–3 in Fig. 2a), indicating the presence of an attractive force gradient exceeding the cantilever stiffness (~ 0.2 nN nm^−1^). Then the tip was pushed further to contact the substrate from point 3–4 until the contact ramp. A maximum loading force of 5 nN was applied with a dwell time of 2 s to stabilize the interaction. Tip retraction was performed (point 4–5) via different loading rates and was always terminated by a larger jump-from-contact event (point 5–6) consistent with strong attractive interaction. To estimate the equilibrium rupture force (*f*_*eq*_), sets of force curves at five different pulling rates were collected and *f*_*eq*_ at each azimuthal orientation was obtained by fitting the data to the multiple bond model of Friddle et al.^29^.

Figure 2b shows the measured jump-from-contact forces at different azimuthal orientations from 0° to 120° for a selected tip area (381 nm diameter). Because the crystallographic orientation of the *a* and *b* axes of the ZnO substrate was pre-determined from single crystal X-ray diffraction (Supplementary Fig. 7), and because the same was known for the tip based on its ZnO nanowire morphology, measurements could be performed based on absolute azimuthal orientations (Supplementary Note 3). The force decreases with azimuthal orientation progressing from 0° to 30° for each pulling rate, then increases with azimuthal orientation progressing from 30° to 60°. Another force minimum and maximum are obtained upon progressing from 90° to 120°. For comparison, the interaction force between the ZnO(0001) tip and a flat silicon wafer substrate bearing the usual amorphous silicon oxide passivation layer was also measured (Fig. 2b); in this case the interaction is rather weak and no periodicity is observed, as expected. Figure 2c shows the fitted curves for azimuthal orientations from 0° to 60°. All resulting equilibrium interaction forces are plotted in Fig. 2d and clearly display a cyclic nature with a repeat period of 60° and force maxima at 0°, 60°, and 120°. To approximate the average maximum and minimum rupture force, the data were fit using a simple sinusoidal waveform (Methods section). A maximum rupture force of 79.78 nN was obtained when the azimuthal orientation is 0°, 60°, and 120° and a minimum rupture force of 47.30 nN was obtained when the azimuthal orientation is 30° and 90°. The maximum and minimum interfacial forces thus occur in a repeating manner that is twice the bulk crystallographic periodicity of the ZnO wurtzite crystal structure along the *c* axis.

**Fig. 3:**
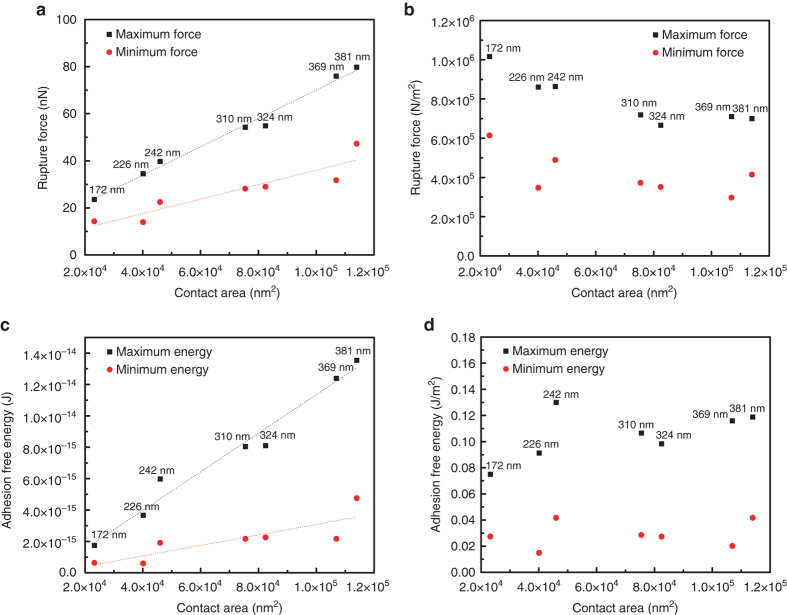
Maximum and minimum rupture forces and adhesion-free energies of ZnO(0001)–ZnO(000$\bar 1$). **a** Force versus tip contact area; **b** force normalized to tip contact area; **c** the corresponding adhesion-free energy versus contact area; **d** adhesion-free energy normalized to tip contact area. Tip diameters are noted

The effect of contact area on the interaction force was investigated using tips fabricated with systematically varying diameters ranging from 381 nm down to 172 nm (Fig. 3). In all cases, the derived equilibrium interaction force exhibits cyclical dependence on azimuth with a period of 60° (Supplementary Fig. 8). The determined maximum and minimum rupture forces for seven different contact areas are plotted in Fig. 3a, which show a linear dependence on contact area. When normalized to the tip surface area, the maximum and minimum rupture forces are approximately constant, showing a difference of around 5 × 10^5^ N m^−2^ (Fig. 3b). This result supports our conclusion that face-to-face contact between the ZnO(0001) AFM tips and the ZnO(000$\bar 1$) substrate was generally achieved during the force measurements.

The energy required to overcome the stiffness of the AFM cantilever when it reaches *f*_eq_ is given by^40^:

1 $$E = \frac{{f_{{\rm{eq}}}^2}}{{2{k_{\rm{c}}}}}$$

where *k*_*c*_ is the cantilever spring constant. The value *E* corresponds to the free energy difference between the bound and unbound states, and is thus the adhesion-free energy. The value *E*/2 is the interfacial free energy per ZnO surface, averaged for the two different terminations. The resulting adhesion-free energies show a linear dependence on the tip contact area (Fig. 3c), and after area normalization the maximum and minimum adhesion-free energy are obtained (Fig. 3d). The maximum values are more than an order of magnitude lower than the cleavage energy of ZnO along the *c* axis (~ 4.0 J m^−2^)^41^, which is the theoretical upper limit, consistent with the likelihood that some of the waters of hydration remain in the interfacial region during contact, as discussed below. Corresponding interfacial free energies (~ 0.015–0.053 J m^−2^) are also substantially lower than measured hydrated surface enthalpies of ZnO nanocrystals (~ 1.3 J m^−2^)^42^, consistent with the clear difference in our measurements that hydration water is confined between the two ZnO surfaces. The difference between the maximum and minimum adhesion-free energy was regularly ~ 0.076 J m^−2^.

### Molecular simulation of ZnO(0001)–ZnO(000$\bf \bar 1$) interactions

To have a better understanding of the origin of the observed angular dependence of the interaction, we carried out PMF calculations for ZnO(0001)–ZnO(000$\bar 1$) solvated in water using empirical atomistic force fields and MD simulations (Methods section). In the simulation system the (0001) termination of a hexagonal ZnO nanoparticle faces the (000$\bar 1$) termination of a 2-D ZnO slab, surrounded by water molecules. In the simulations, the orientation and position of the substrate are fixed while the particle is placed in different azimuthal orientations with respect to the substrate (0–120° with an interval of 15°) and the distance dependence of the PMF is computed at each orientation.

**Fig. 4:**
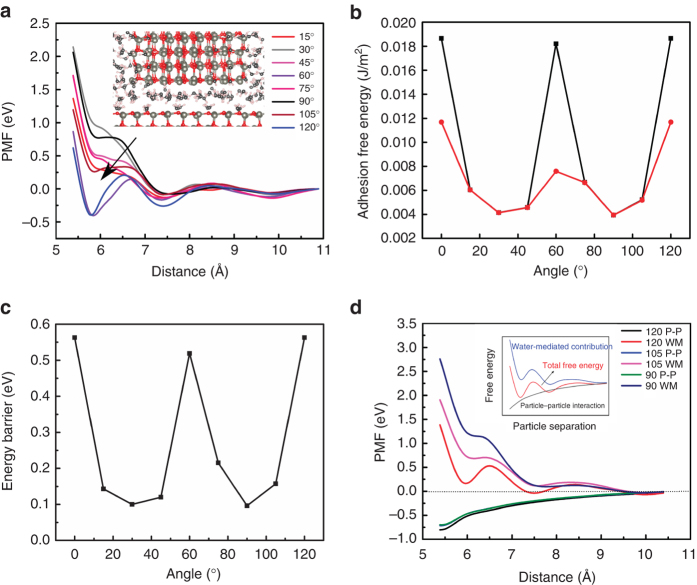
Calculated PMF curves and energy barriers for ZnO(0001)–ZnO(000$\bar 1$) interactions at different azimuthal angles. **a** PMF curves at different azimuthal angles. An exemplary configuration at the first energy well is also shown. **b** Adhesion-free energies between ZnO particle and the substrate by using global minima (*black filled circles*) and second minima (*red filled circles*), at different angles. For better comparison with the adhesion-free energies estimated from experiments as shown in Fig. 3d, the free energies obtained from **a** are normalized to the surface area and the unit of J m^−2^ is used. **c** Energy barriers for moving the particle out of the lowest energy minima at different angles. **d** Comparison of contributions to the total PMF from particle–particle (P–P) interactions and from the water-mediated forces at 90°, 105°, and 120°. The total PMF and the P–P interaction contribution are calculated from MD simulations, and the water contribution is obtained as a difference between the previous two terms as shown in the inset

The resulting PMF curves at all azimuthal angles share a common feature. That is, there are three free energy wells at ~ 5.8, ~ 7.5, and ~ 9.7 Å, respectively (Fig. 4a). The first energy well corresponds to a configuration where there are two water layers in-between the two opposing surfaces. The first layer water molecules are located above vacancies created to remove the surface dipole of the ZnO(000$\bar 1$) slab. In the second layer, the water molecules form a hydrogen-bonded network with both the first water layer and atoms on the ZnO(0001) surface of the particle. Correspondingly, three water layers are found to exist between the two surfaces in the configuration at the second well, and four water layers are associated with the third well.

Based on the number and character of the wells as a function of azimuthal orientation, the PMF curves can be divided into three groups (one exemplary force curve and PMF curve of each group are shown in Supplementary Fig. 9). The first group is composed of the PMFs at 0° (or equivalently 120°) and 60°, in which the energies at the first well are strongly negative (−0.36 to −0.40 eV) and much more attractive in character than at any other wells found in the system. In the second (15°, 45°, 75°, and 105°) and third (30° and 90°) groups, the energies at distances corresponding to this first well are positive but distinct in terms of how positive, with the latter being more positive than the former. The global minimum for both groups 2 and 3 is at a distance corresponding to the second energy well of the first group.

For comparison with the orientation dependence of the adhesion-free energies determined by DFS, we computed corresponding values from the MD simulations, taken as the difference between an energy minimum and zero energy at a far separation for each azimuthal angle, normalized to interfacial area (Fig. 4b). If we consider the second well, where three layers of intervening water persist at all azimuthal angles (Fig. 4b, red line), a periodicity of 60° is evident, identical to that observed from the force measurements. This periodicity, again occurring as twice the bulk crystallographic periodicity, can now be clearly identified as corresponding to the period of dominant alignment of opposing surface cation and anion sub-lattices across the solvated interface. At 0° and 120° the two lattices are in symmetry equivalent bulk crystallographic alignment along the *c*-axis. At 60°, one lattice has a translation of $1{\rm{/3}}\left\langle {01\bar 10} \right\rangle$ with respect to the other, and thus the interface resembles the very common type-I stacking fault with a stacking sequence of (…AaBbAaCcAaCc…) in the wurtzite structure^43^. This type of stacking fault has been shown to have a very low formation energy^44^. As one major finding from the simulations, it appears that this low-energy configuration manifests even across the solvent-filled gap.

Calculated maximum and minimum adhesion-free energies as a function of azimuth generally compare remarkably well with those determined by DFS. For the second well they are lower by a factor of approximately 10. However, if the global minimum well at each angle is considered, which includes the first well for crystallographically aligned angles (Fig. 4b, black line), the agreement improves to a factor of 5. The adhesion-free energy is substantially larger when the two surfaces are aligned, where only two water layers are found to persist between them. In this case, the adhesion-free energy difference per contact area between 60° or 120° and 30° or 90° (~ −0.015 J m^−2^), as well as the relative ratio between these two values (~ 4.5:1), show the best correspondence with the DFS data. The analysis strongly suggests that in the measurements, the deepest well for each angle is always achieved; the nanocrystal tip reached the first well when aligned at 60° or 120°, and the second well at the rest of the angles. The 60° periodicity continues to hold true regardless of which well is considered, and the overall correspondence between the experiments and simulation for the azimuthal dependence of the adhesion-free energy is striking.

Analysis of the PMF calculations can be extended to consider the energy barriers that correspond to moving the particle out of its global minimum to larger separations at each azimuthal angle (Fig. 4c). The azimuthal dependence of this detachment free energy barrier also shows a periodicity of 60°, with maximum values corresponding to aligned conditions and minimum values at misaligned conditions. With respect to an oriented aggregation process, these barriers suggest it is easier for nanoparticles attracted in a solvent-separated state to detach from each other when misaligned compared to when they are aligned, consistent with a higher probability of interparticle rotation when misaligned, maintaining the search for perfect lattice alignment. The inverse analysis of barriers for moving the particle from the second to the first well is somewhat precluded by the lack of a first minimum for misaligned angles (Fig. 4a), but by inspection of the PMFs these barriers would clearly be lowest for aligned conditions, consistent with a higher probability of attaining shorter interparticle separations when alignment is achieved.

To better understand the origin of this free energy landscape, we also performed PMF calculations in vacuum to compute solvent-independent contributions to particle–particle (P–P) interaction free energies at 90°, 105°, and 120°, each representative of its own group (Fig. 4d). At all three angles, the PMF curves decrease monotonically with decreasing separation within the same distance range as that which we explored in the solution calculations. At ~ 5.8 Å, the energy at 90° is very close to that at 105° (~ 0.01 eV), and thus both are about 0.05 ~ 0.06 eV higher than that at 120°. These small differences are not enough to account for the much larger difference in energy at the first energy well between group 1 and the other two groups and neither for the difference between group 2 and group 3.

The water-mediated contribution to the free energy was obtained (as shown in the inset of Fig. 4d) as a difference between the total PMF in solution and the PMF in vacuum containing only P-P interactions. From the analysis of water contribution alone it immediately becomes clear that the water-mediated forces play a decisive role in determining the shape of free energy curves as well as the angular dependence of interfacial interactions in our simulation system. The free energy oscillations with distance show a period of about the diameter of a water molecule, mimicking the well-known oscillatory solvation force that can occur between two solid surfaces at small separations (<a couple of nm)^45^. On the other hand, the difference in water-mediated forces, similar to the repulsive hydration force between two hydrophilic surfaces^45–47^, at different angles might be the origin of the orientation dependence of the interactions that emerge from our simulations, and by similarity the measured rupture force. The theory of hydration repulsion remains poorly understood due to its complex involvement of water-water interaction, water-surface interaction, and entropic contributions^46^. Our analysis of hydrogen bonds in-between the two ZnO surfaces shows (Supplementary Note 4) no obvious angular dependence on the number of hydrogen bonds and the hydrogen bond length (Supplementary Fig. 10). But we find a similar cyclic change with a period of 60° in the number of Zn-O_water_ bonds in the space between the two ZnO surfaces (Supplementary Fig. 11).

From this study, we find that classical MD/PMF calculations reproduce with striking similarity the same period of angular dependence of ZnO-ZnO interfacial interaction as that observed in the rupture forces measured in solution. The complete correspondence reveals the prominent role of water structuring for “communicating” azimuthal alignment between like surfaces across the inter-surface, or in the case of oriented attachment inter-particle, solvent gap. Water structuring also creates attachment barriers that preferentially maintain the solvent-separated state during misalignment. The observed angular dependence of adhesion therefore arises less from P–P interactions and more from water-mediated forces that oscillate with mutual crystallographic alignment. The findings indicate that direction-specific interaction forces can create torque to align ZnO nanocrystals at close separations to induce the oriented attachment, which can explain the ex situ TEM observations that ZnO nanoparticles can aggregate via oriented attachment to form rod-like single crystals. The findings also can help to explain the oriented attachment behaviors of other material systems and understand the self-assembly of nanocrystals, Schiller layer formation, and even the grain boundary construction in polycrystals.

## Methods

### Force measurement

The studies were performed using an Asylum MFP-3D AFM. The experimental setup is shown in Supplementary Fig. 1. Atomically flat and oriented face-specific single crystals to be used as ZnO substrates were adhered to a flow chamber equipped with a rotation stage using Crystalbond™ 509 Mounting Adhesive (Structure Probe, Inc.). Another atomically flat and oriented face-specific single nanocrystal was loaded onto an AFM cantilever, using nanofabrication methods described in the following Methods section. The lattice orientation of the ($000\bar 1$) ZnO substrates (i.e., the <$10\bar 10$> directions within the surface plane) was determined using x-ray diffraction and the lattice orientation of the (0001) ZnO AFM tip was determined using SEM (Supplementary Note 2). The azimuthal angles were adjusted by rotating the ($000\bar 1$) ZnO substrates in the fluid cell. The stability of the (0001) ZnO AFM tip was investigated in detail via using ex situ SEM (Supplementary Note 1) and the surface stability of the ZnO(000$\bar 1$) bulk substrate was detected using in situ liquid cell AFM (Supplementary Movie 2).

### Nanoscale ZnO(0001) AFM tip fabrication

Fabricating oriented face-specific ZnO(0001) nanosized AFM tips with atomically flat clean surfaces is a challenge. Beyond the difficulty of achieving these characteristics, the (0001) tip face must also be co-planar with the substrate surface throughout force measurements and stage rotation in order to maintain a known area of interaction. Among several fabrication methods the most tried is by glue-mounting single crystals using macroscopic techniques, which due to the difficulties of manipulation and orientation control is usually limited to micron-sized particles^48^. Another method is to grow materials on original AFM tips using physical vapor deposition (PVD), chemical vapor deposition (CVD), ion beam sputter (IBS) deposition, or electrodeposition^49^, but this invariably leads to polycrystalline materials. The method used in the present study is microscopic manipulation and mounting of oriented single ZnO nanowires (NWs) onto AFM cantilevers that are then downsized further to a controlled nanoscale surface area by focused ion beam (FIB) milling^50^. To avoid contamination of the tip surface by ion impact^51^, special care was required (Supplementary Fig. 4).

ZnO NW arrays expressing hexagonal prismatic surfaces were synthesized on gold substrates via a hydrothermal method (Supplementary Fig. 4a)^52^. The method produced NWs with the exposed upper wire surface as the Zn-terminated (0001) face^53^. Individual ZnO NWs were nanomanipulated onto AFM cantilevers (Omniprobe, Oxford Instruments) inside of a SEM for real-time observation (Helios NanoLab 600i, FEI, Hillsboro, OR). For the specific AFM used for the force measurements, the cantilever was mounted with an 11° tilt from the substrate, so the AFM tips onto which the ZnO NWs were mounted were first pre-milled to compensate for this tilt angle (Supplementary Fig. 5). To securely hold the ZnO NWs on the cantilever, a hole was then drilled into the tip, orthogonal to its tilted milled surface (Supplementary Fig. 4b). To transfer a ZnO NW onto the cantilever, the Omniprobe was driven close to a selected ZnO NW and then a rectangular patch of platinum (Pt) (50 nm thick) was deposited on its side surface by ion-beam-assisted deposition (Supplementary Fig. 4a), which enabled it to then be picked up by the Omniprobe, relocated and oriented over the AFM tip prepared for mounting, manipulated into the hole, and finally secured with Pt (Supplementary Fig. 4c). FIB milling at high voltage (30 kV) with Ga ions was used to remove any excess Pt present on the top and side surfaces and to sharpen the ZnO NW into a needle-like shape (Supplementary Fig. 4d). Once prepared, the likelihood of Pt and Ga contamination^51^ was mitigated first by FIB milling at low voltage (2 kV), to reduce the thickness of the damaged zone, and then by polishing the ZnO NW tip by high-speed scanning in the AFM^54^ (Supplementary Fig. 4e). SrTiO_3_ with a high density of nanoparticles was used as the polishing substrate; the height and the size of the nanoparticles were less than 8 and 50 nm, respectively. The resulting ZnO(0001) AFM tips were atomically flat and oriented, which was confirmed by both SEM and reverse imaging AFM (Fig. 1). EDX analysis detected only Zn and O on the tip surface (inset in Fig. 1b). This fabrication method yielded a series of ZnO NW tips exposing systematically varying nanoscale areas of the (0001) face so that the contact area dependence of the interfacial force could be determined.

The effective miscut angle of the ZnO substrate as given by the supplier was ~0.2 degrees. The effective miscut of the ZnO tip, on the basis of the step density observed in reverse AFM imaging, was also approximately 0.2 degrees. These are as good as practically achievable on this material.

### Analysis of DFS data

The DFS data was analyzed using the generalized multiple bonds model proposed by Friddle, Noy, and De Yoreo^29^. This model provides a comprehensive description of force spectra for a diverse suite of bonds over experimentally relevant pulling rates. The interaction force between two crystal faces is given by:

2 $${\left\langle f \right\rangle _N} = \,{f_{{\rm{eq}}}} + N{f_\beta }{e^{\frac{N}{{R({f_{{\rm{eq}}}}{\rm{/}}N)}}}}{E_1}\left[ {\frac{N}{{R({f_{{\rm{eq}}}}{\rm{/}}N)}}} \right]$$

3 $$R\left( {{f_{{\rm{eq}}}}{\rm{/}}N} \right) = \frac{r}{{{k_{\rm{u}}}\left( {{f_{{\rm{eq}}}}{\rm{/}}N} \right){f_\beta }}}$$

where *f*_eq_ is the equilibrium force for the bond/transducer system, *r* is the loading rate, N is the equilibrated number of formed bonds at zero force, ${f_\beta } = \frac{{{k_B}T}}{{{x_t}}}$ is the thermal force scale, *k*_*B*_ is Boltzmann’s constant, *T* is the temperature, *x*_*t*_ is the barrier location, ${E_1}(z)\, = \,{\int}_z^\infty {\frac{{{e^{ - s}}}}{s}{\rm d}s}$ is the exponential integral, *r* is the pulling rate, ${k_u}(f) = k_u^0{e^{\beta (f{x_t} - 0.5{k_c}x_t^2)}}$ is the unbinding transition rate, *k*_*c*_ is the spring constant of the AFM cantilever and the $k_u^0$ is the intrinsic unbinding rate.

The resulting equilibrium interaction forces for different azimuthal orientation were fit using the sinusoidal waveform:

4 $${f_{{\rm{eq}}}} = {f_0} + A \times {\rm{sin}}\left( {2\pi \frac{{x - xc}}{\omega }} \right)$$

where *f*_0_ is the offset, *A* is the amplitude, *x* is the azimuthal orientation, *xc* is the phase shift, and the *ω* is the period.

### Classical MD simulations

Classical MD simulations were performed at room temperature and zero-applied pressure by using DL_POLY Classic^55^. The calculations were conducted in the NVT ensemble using the Nosé-Hoover thermostat^56^. The equations of motion were integrated using the Verlet-leapfrog algorithm with a time step of 2 fs. The Ewald sum method was used to calculate the long-range Coulombic interactions with a real-space cutoff of 15 Å. The same cutoff distance was used for the short-range interactions. The force fields used in this study are described in the Supplementary Table 1. The ZnO substrate had dimensions of approximately 59 × 51 × 24 Å^3^ and it consisted of 10 Zn-O double layers, with the middle four layers fixed. 25% of the surface Zn or O atoms on the respective surface of the ZnO substrate were removed in order to eliminate the net dipole normal to the (0001) surfaces. The ZnO nanoparticle consisted of seven Zn-O double layers and a (0001) surface area of about 340 Å^2^. No vacancies were introduced on the (0001) and (000$\bar 1$) surfaces of the ZnO particle as zero net dipole was predicted by METADISE^57^. Steps on basal surfaces of both the nanoparticle and the substrate were not included in our simulations since there was no obvious four-fold symmetry in the rupture force data and thus the step perturbation is trivial. The water slab had a density of ~ 0.96 g/cm^3^. In order to keep the surface Zn atoms of the bottom substrate from dissolving into the solution, a harmonic tethering potential was applied to those Zn atoms (Supplementary Table 1). In PMF calculations (Supplementary Note 5), the orientation of the substrate was kept constant while the nanoparticle was initially rotated from 0° to 120° with an interval of 15°. At each angle, a system with two surfaces separated by two water layers in solution was run for 2 ns (Supplementary Movie 3), whose output was used for the starting structure for further PMF calculations in respective orientation case. In each PMF calculation, the system was first equilibrated for 200 ps and then was run for 3 ns for production. The force to constrain the particle along the normal to the basal plane was collected for the last 1.2 ns. In cases where the force was not converged within the first 3 ns, the simulations were extended for another 2 ns to ensure convergence. The hydroxylation effect on the surface water structure was assessed in Supplementary Note 6 and Supplementary Fig. 12.

### Data availability

Data supporting the conclusions presented in this study are available from corresponding author upon request.

## Electronic supplementary material

Supplementary Information

Description of Additional Supplementary Files

Supplementary Movie 1

Supplementary Movie 2

Supplementary Movie 3
