# Supplementary material for: Direction-specific interaction forces underlying zinc oxide crystal growth by oriented attachment
Source: Nat Commun. 2017 Oct 10;8:835. doi: 10.1038/s41467-017-00844-6 (PMC5635138; doi:10.1038/s41467-017-00844-6)
Supplement: Supplementary file 1 — Supplementary Information [file 41467_2017_844_MOESM1_ESM.pdf]

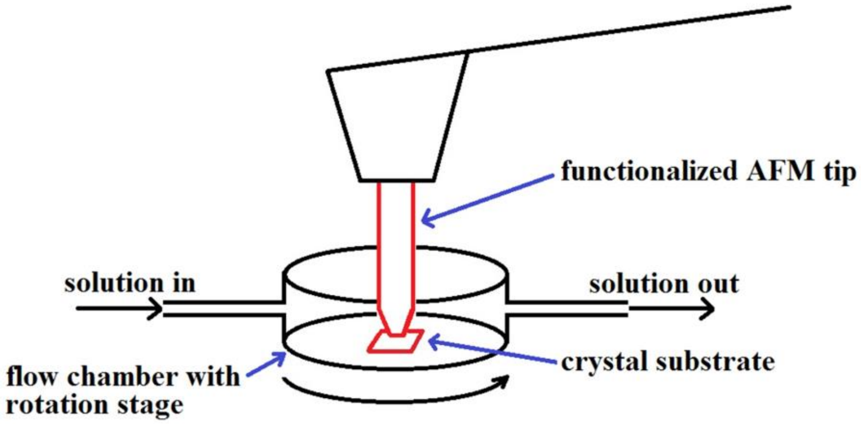

Supplementary Figure 1. Schematic of the force measurement experiment.

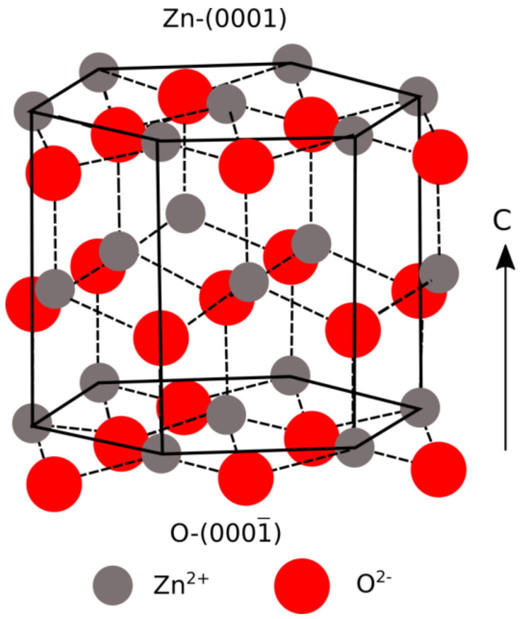

Supplementary Figure 2. Structure model of ZnO.

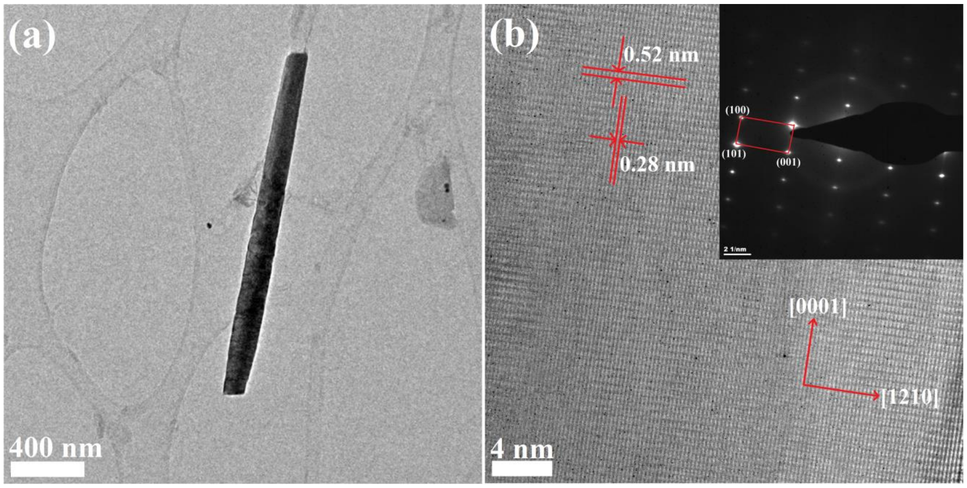

Supplementary Figure 3. TEM (a) and HRTEM (b) images of synthezied ZnO nanowires. The insert in (b) is the SAED pattern of the ZnO nanowire.

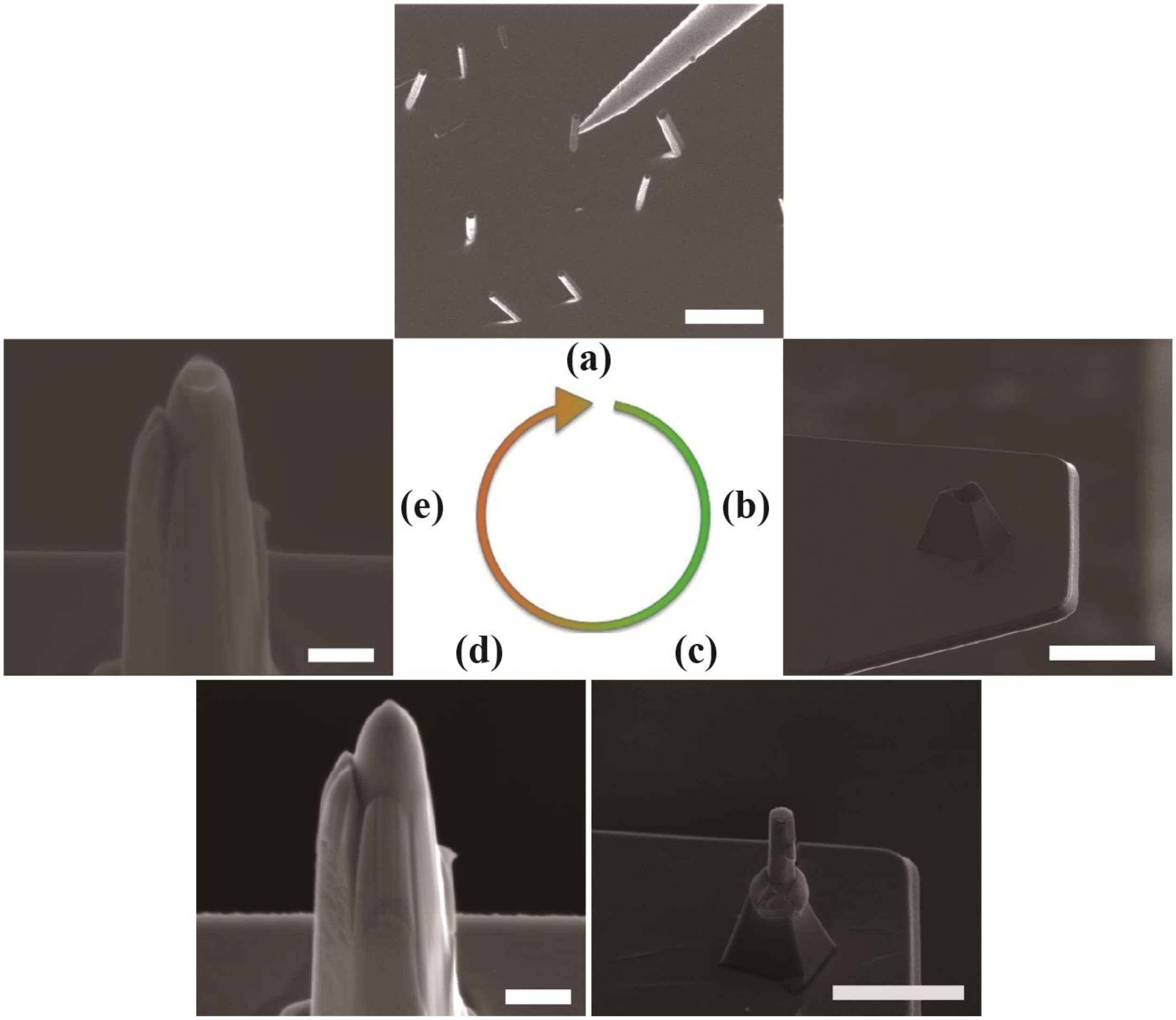

Supplementary Figure 4. Procedure for fabricating oriented nanoscale ZnO(0001) AFM tips. **(a)** Binding a ZnO NW onto the Omniprobe (scale bar 5 µm); **(b)** FIB milled hole in pre-milled AFM tip (scale bar 5 µm); **(c)** ZnO NW secured by Pt on the AFM tip (scale bar 5 µm); **(d)** The ZnO (0001) AFM tip after FIB sharpening (scale bar 0.5 µm); **(e)** The ZnO (0001) AFM tip after high-speed polishing using AFM (scale bar 0.5 µm).

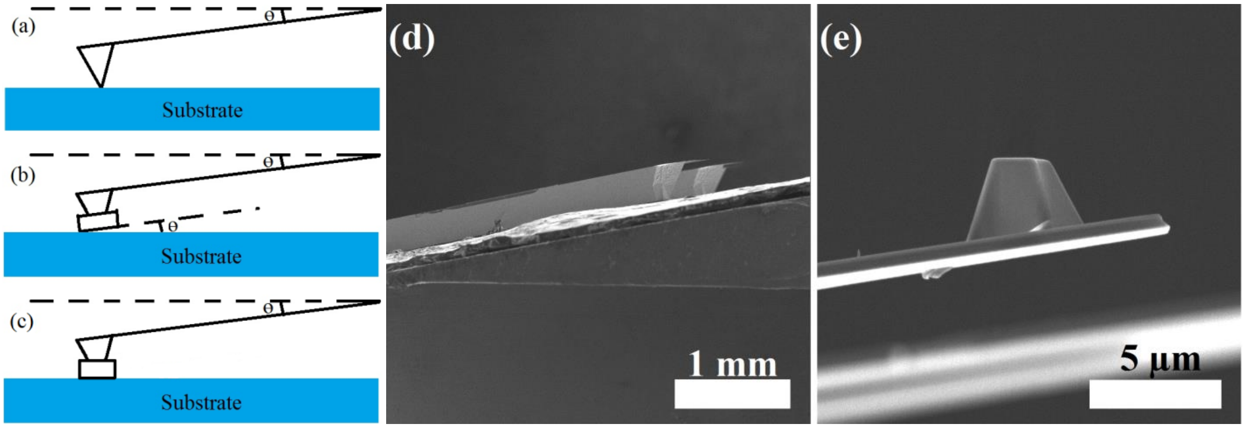

Supplementary Figure 5. The original AFM tip cutting strategy. The cartoon images of **(a)** original AFM tip, **(b)** FIB cut AFM tip without tilt angle correction, and **(c)** with tilt angle correction; **(d)** and **(e)** SEM images of the AFM tip glued on a stage with 11° tilt for adjusting the AFM tip surface to the horizontal plane during tip fabrication.

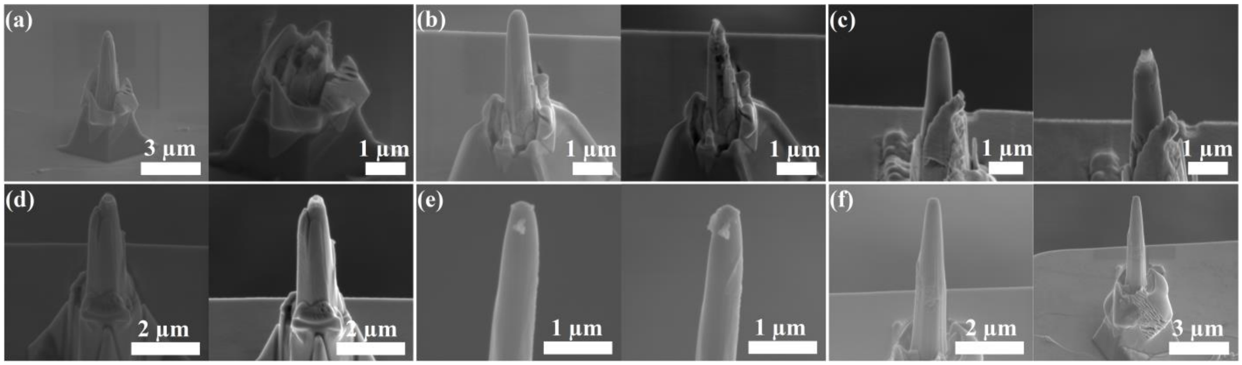

Supplementary Figure 6. SEM images of ZnO (0001) AFM tips before (left tip in each image) and after (right tip in each image) force measurement in different flowing solutions: **(a)** pure water (8 h), 0.01 mM **(b)**, 0.05 mM **(c)**, 0.1 mM **(d)**, and 1.0 mM **(e)** $Zn(NO_3)_2$ solution (8 h), and **(f)** 0.2 mM $Zn(NO_3)_2$ solution (16 h).

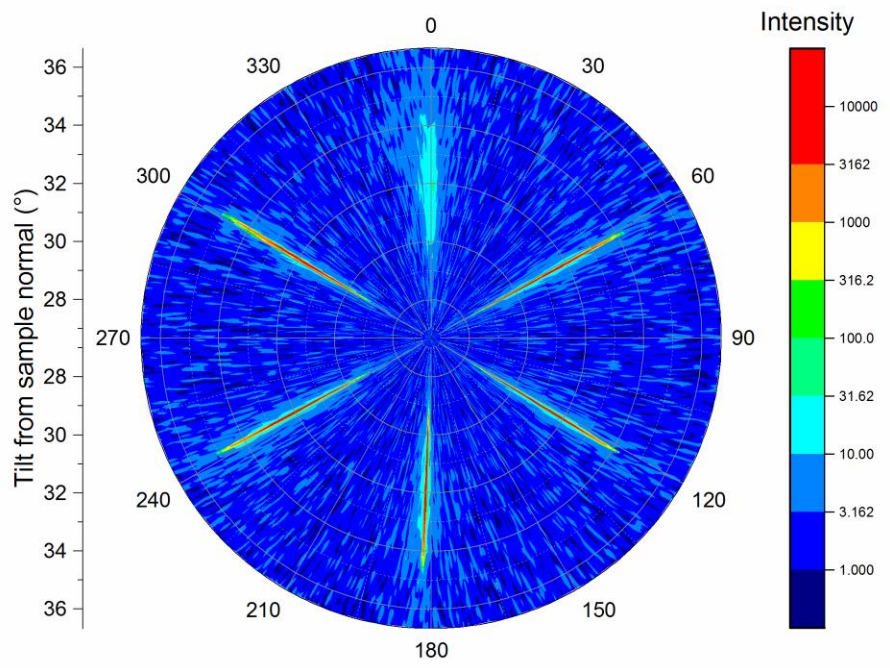

Supplementary Figure 7. Intensity of ZnO ($10\bar{1}3$) diffraction peak. The diffraction peak was plotted for the scattering vector at different rotational angles to a marked direction along the sample surface (circumferential axis) and the tilt to the surface normal (radial axis).

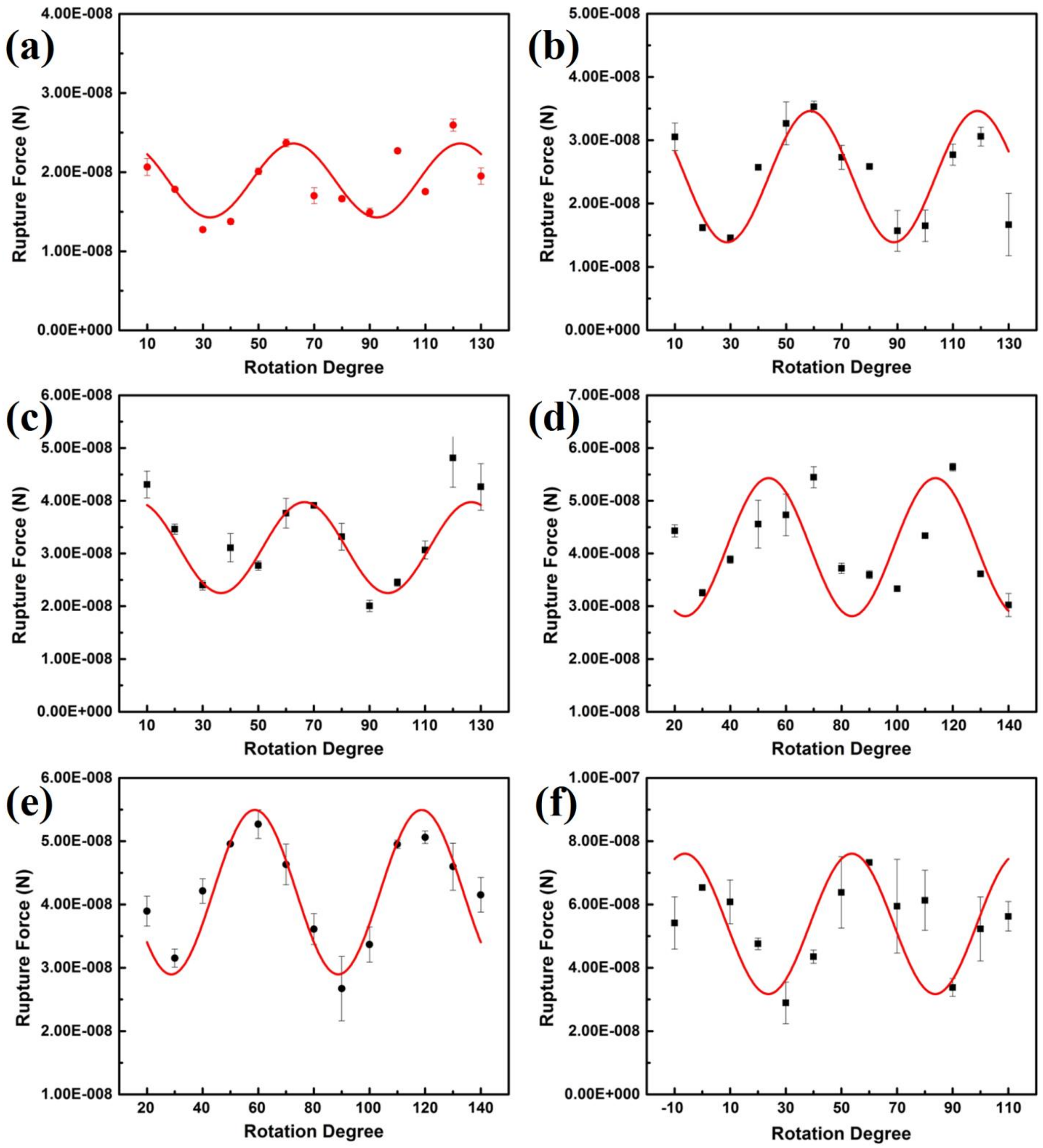

Supplementary Figure 8. Rupture forces at different azimuthal orientation with different tip diamters, showing best sinusoidal fits. (a) 172 nm; **(b)** 226 nm; **(c)** 242 nm; **(d)** 310 nm; **(e)** 324 nm; and **(f)** 369 nm. Error bars derived from the fitting of the multiple bond model.

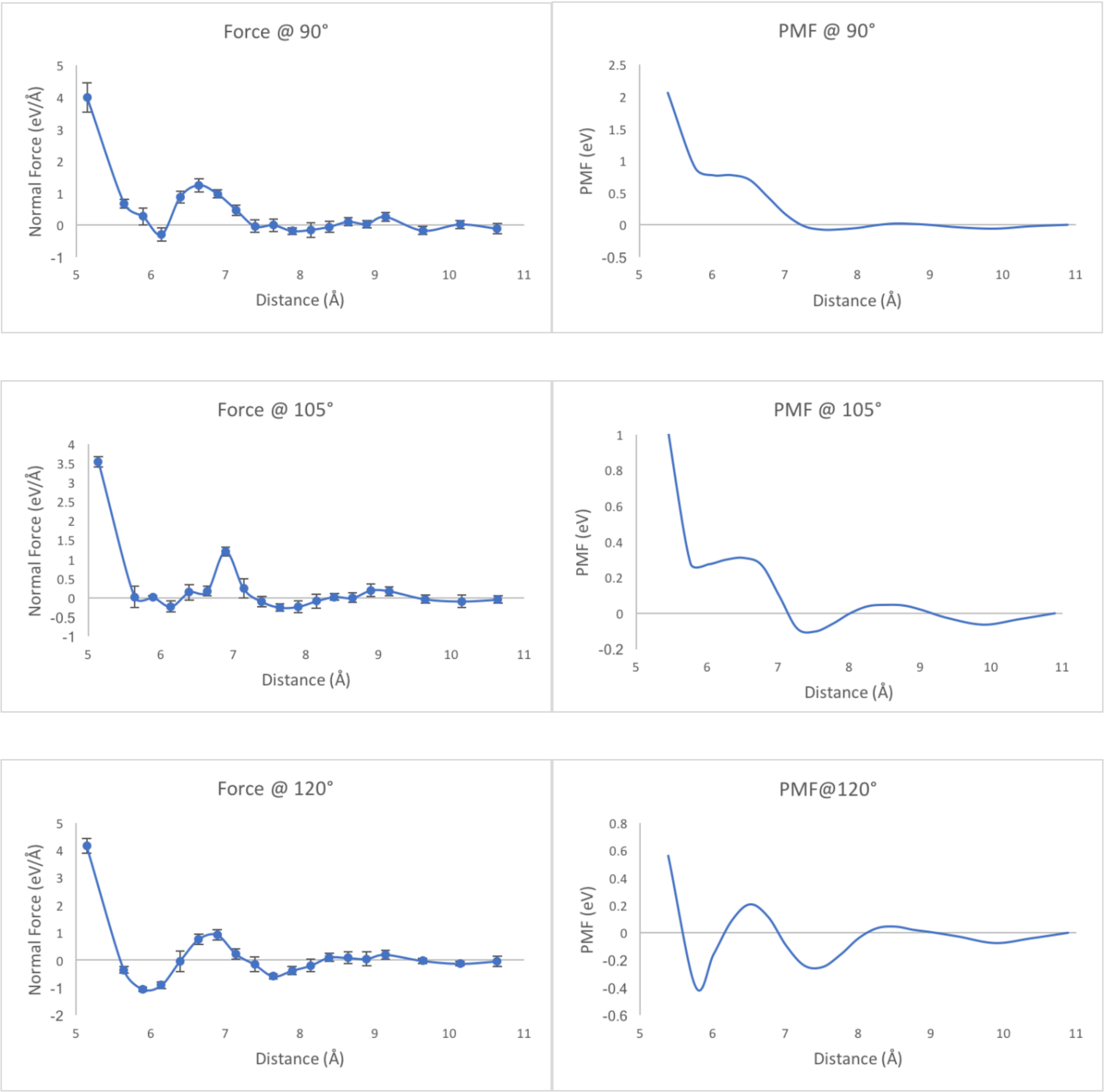

Supplementary Figure 9. Exemplary force curves and PMF curves at 90º, 105 º, and 120º. The force data at each separation and angle were grouped into ten groups, and the standard deviation of the averages of the ten groups was used as the error bar for each data point shown in the force curves.

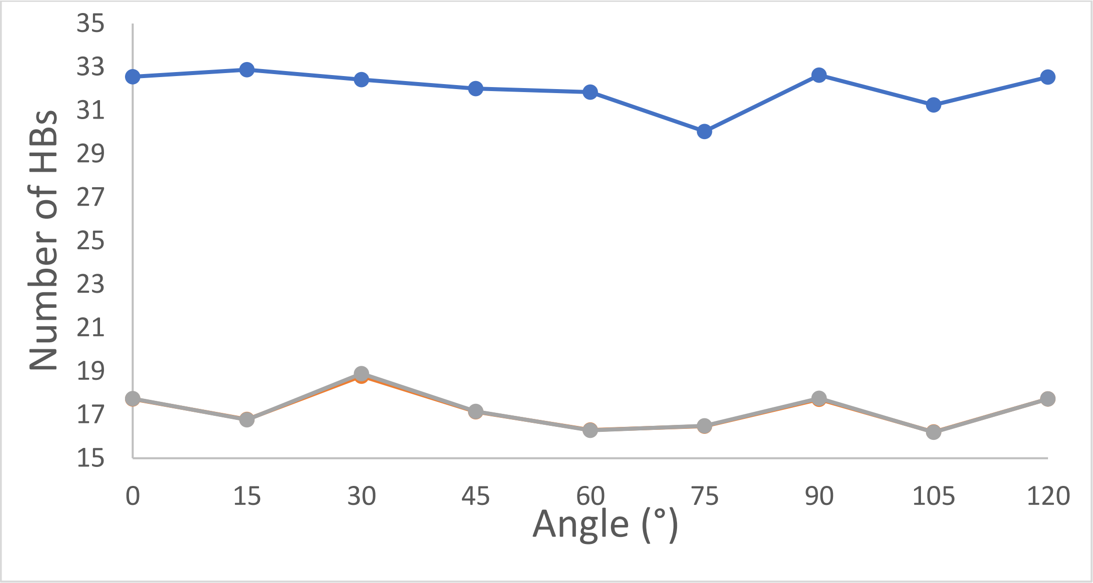

Supplementary Figure 10. Number of hydrogen bonds at different angles. Total numbers of hydrogen bonds in between two ZnO surfaces (blue line for radius cutoff of 10 method); and number of hydrogen bonds between nanoparticle surface O and nearby water (red line for radius cutoff of 10 method and gray line for edge atom exclusion method).

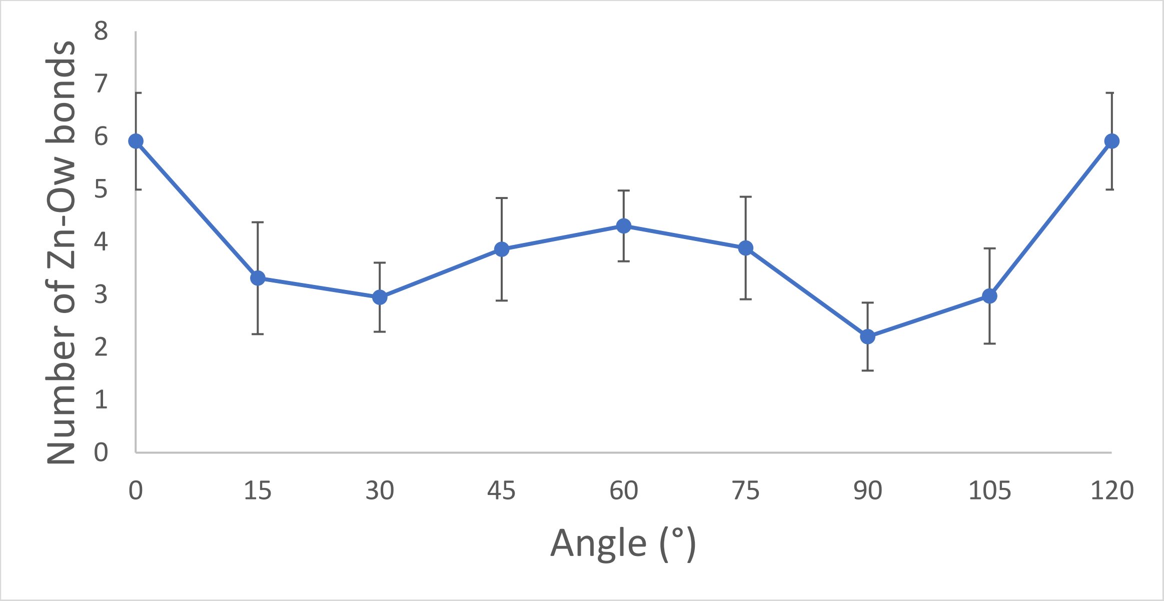

Supplementary Figure 11. Number of Zn-Ow bonds in between nanoparticle surface Zn and nearby water molecules at different angles. The numbers of bonds at each angle were grouped into ten groups, and the standard deviation of the averages of the ten groups was used as the error bar for each data point shown in the force curves.

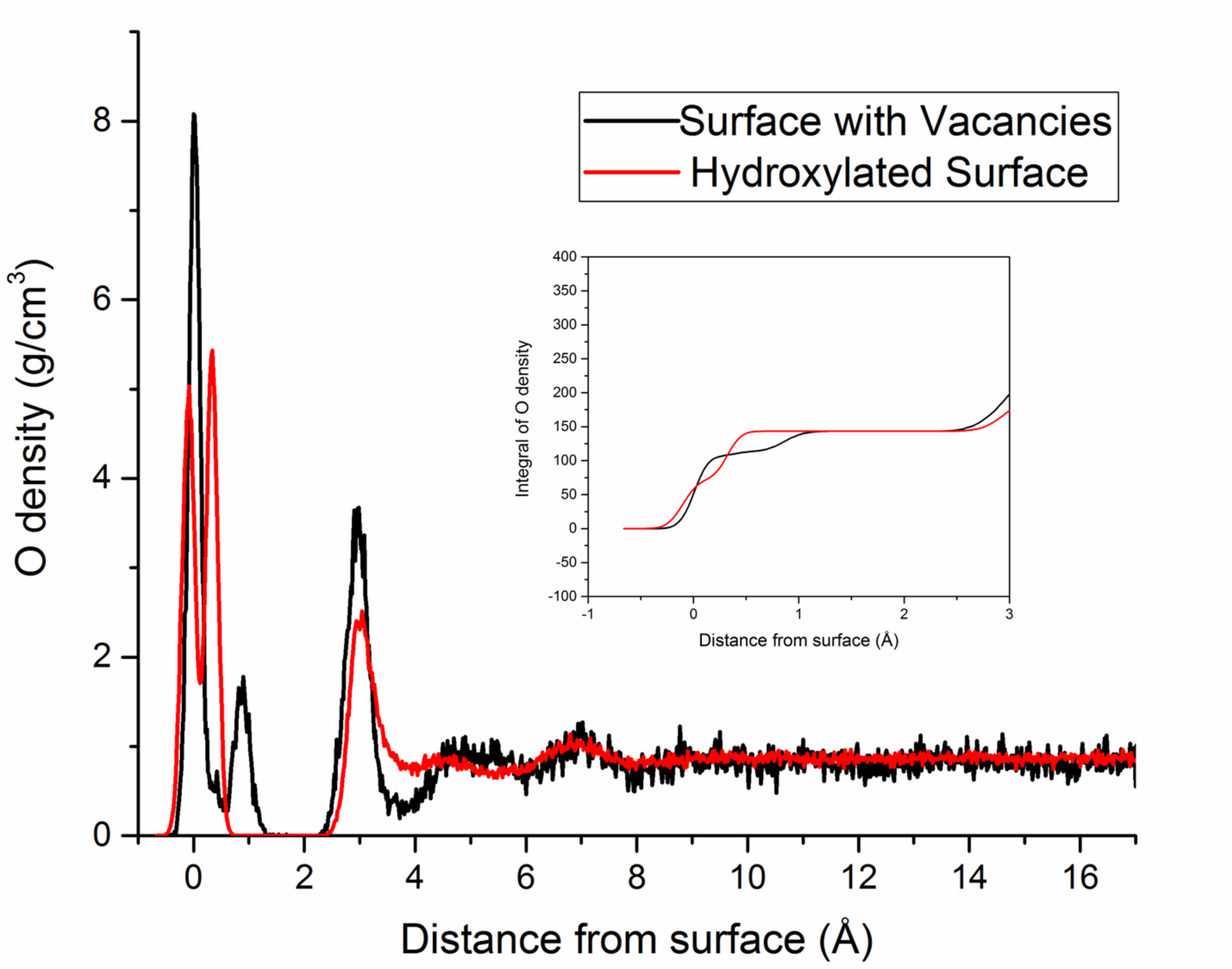

Supplementary Figure 12. Oxygen atom density profile above H-(1×2) ZnO (000$\bar{1}$) surface (red curve) and surface with isolated vacancies (black curve). The integrals of the first two peaks for two surfaces are shown in the inset.

Ionic Species:

Ion	Charge (e)	Mass (g/mol)	Note
Zn	+2	65.39	Zn in ZnO
O	-2	15.999	O in ZnO
OW	-0.8476	15.999	O in H_2O
H	+0.4238	1.008	H in H_2O

Intermolecular Interactions: Buckingham potential $U(r) = Aexp\left(-\frac{r}{\rho}\right) - \frac{C}{r^6}$

Ion pair (ij)		A(eV)	ρ(Å)	C (eV•Å^6)
Zn	O	529.70	0.3581	0.0
O	O	9547.96	0.21916	32.0
Zn	OW	14974.51	0.186012	0.0

Lennard-Jones potential $U(r) = \left(\frac{A}{r^{12}}\right) - (\frac{B}{r^6})$

Ion pair (ij)		A (eV•Å^{12})	B(eV•Å^6)
OW	OW	27329.82	27.1275
O	OW	110568.00	27.6545

Tethering potential: $U(r) = \frac{1}{2}\, kr^2$ where *r* is the distance of the atom from its position at time *t* = 0. k = 0.8 eV/Å^2 was used for surface Zn atoms.

Supplementary Table 1: Potential parameters used in this work. A rigid-ion model (RIM) version of the model of Binks and Grime was used for ZnO[1]. The SPC/E model[2] was used to simulate water molecules. Constraints in SPC/E water model: r_{OH}=1.000 Å, r_{HH}=1.63298 Å. The interactions between ZnO and water were described by two interatomic potentials, that is, a Buckingham potential for Zn-O_{water} and a Lennard-Jones potential for O_{oxide}-O_{water}. The parameters for the former were taken from Harris et al.[3]. The parameters for the latter were fitted to optimize the agreement with the geometries and adsorption energies of molecular water adsorption on $(10\bar{1}0)$ and $(11\bar{2}0)$ surfaces from density functional theory (DFT) calculations[4,5].

Supplementary Note 1: Synthesis of ZnO nanowires (NWs) on gold substrates[6]

ZnO NWs to be used as AFM tips were grown on gold substrates, which were made by ion-beam sputter coating 100 nm gold on silicon (100) wafers. A thin layer of Ti (around 10 nm) was deposited on silicon wafers prior to gold deposition. The introduction of a Ti thin film can increase the adhesion of gold thin films with silicon wafers. Before coating Ti and gold thin films, silicon wafers were diced into 1.0 cm × 1.0 cm pieces and treated with Piranha solution (98% H_2SO_4 and 30% H_2O_2 in volume ratios of 3:1) for 30 min at 70 °C, rinsed with de-ionized water and dried with compressed N_2. (Caution: Piranha solution reacts violently with most organic materials and must be handled with extreme care.) The deposition rate for all metals was around 0.2 nm/s. The gold substrates were annealed at 300 °C for 1 h to increase their crystallinity. The gold substrates were stored in desiccators prior to use. The ZnO NWs were synthesized by using a hydrothermal method. The reaction solution was a mixture of zinc nitrate and hexahydrate and hexamethylenetetramine (HMTA). The mole ratio of $Zn(NO_3)_2$ and HMTA was 1:1. The original concentration of these two chemicals was 6 mM. 16 mL of the reaction solution was transferred into the 20 mL Parr bomb. Gold substrates were put face down atop the reaction solution where they remained due to the strong surface tension of the reaction solution. The reaction temperature was 70 °C and the reaction time was 24~48 h. The products were examined using TEM (Supplementary Fig. 3) and SEM (Supplementary Fig. 4a).

Supplementary Note 2: Aqueous solution conditions

We first investigated the effects of solution chemistry (electrolyte type and strength) on the chemical stability of the ZnO(0001) nanocrystal AFM tips. When deionized water was used, dissolution of the (0001) tip surface could be detected by reverse imaging within a few minutes; and the tip was dissolved almost completely down to its mount after 8 h of force measurement trials (Supplementary Fig. 7a). Because the ZnO NWs were synthesized in $Zn(NO_3)_2$ solution, we used $Zn(NO_3)_2$ as the background electrolyte. In micromolar $Zn(NO_3)_2$ concentrations tips were more stable but dissolution could be detected on the hour timescale. For example, Supplementary Fig. 7b, 7c and 7d show a tip partly dissolved after 8 h of force measurements in 0.01 mM, 0.05 mM, and 0.1 mM $Zn(NO_3)_2$ solutions, respectively. At millimolar concentrations deposits formed on the tip; Supplementary Fig. 7e shows deposits formed after 8 h of force measurements in 1 mM $Zn(NO_3)_2$ solution. EDX analysis showed that these deposits were composed of Zn and O, suggesting nucleation and growth of $Zn(OH)_2$ or ZnO at this higher concentration. 0.2 mM was selected for production force measurements, which showed stable tip surfaces up to 16 h (Supplementary Fig. 7f), as well as stable ZnO($000\bar{1}$) bulk substrate surface stability over the same timescale (Supplementary Movie 2).

Supplementary Note 3: Azimuthal angles set up

The lattice orientation of the $(000\bar{1})$ ZnO substrates (*i.e.* the $<10\bar{1}0>$ directions within the surface plane) was determined using x-ray diffraction. The substrate was mounted on the stage of a 4-circle Panalytical MRD diffractometer with its surface normal parallel to the diffractometer sample rotation (phi) axis. The incident beam was monochromatized using a hybrid mirror/4-bounce Ge monochromator and the diffraction angle set to the calculated position for the $(10\bar{1}3)$ peak (62.86°2θ, Cu $K\alpha_1$ radiation). The sample was tilted (through the diffractometer psi axis) so that the scattering vector defined by the incident and detected x-ray paths made a *ca*. 30 degree angle to the surface normal, the calculated angle between the $\{10\bar{1}3\}$ planes and the surface $(000\bar{1})$ plane for a ZnO substrate. Diffracted intensities were recorded in a series of 360° phi scans (*i.e.* full sample rotation) for psi angles between *ca.* 28 and 36°.

The results are plotted on the polar graph shown in Fig.S6. The angle around the circumference represents rotation about the surface normal relative to a directional fiducial mark placed on the surface. The radial axis represents tilting of the sample through the diffractometer psi axis; *i.e.* tilting of the scattering vector away from the surface normal. This figure shows the expected 6-fold symmetry of the ZnO $(000\bar{1})$ surface (*i.e.* we observe the $(10\bar{1}3)$, $(01\bar{1}3)$, $(\bar{1}103)$, $(\bar{1}013)$, $(0\bar{1}13)$, and $(1\bar{1}03)$ planes). The radial elongation of the diffracted intensities arises partly from the scale of the radial axis, and partly from the increased incident beam divergence in this direction. The arrangement of diffraction spots allows us to deduce that one of the ZnO $<10\bar{1}0>$ directions is parallel to the fiducial mark.

The lattice orientation of the (0001) ZnO AFM tip was determined using SEM. As shown in Fig. S4a, the as-synthesized ZnO NW owned hexagonal prismatic surfaces. There are six planes,

including $(10\bar{1}0)$, $(01\bar{1}0)$, $(\bar{1}100)$, $(\bar{1}010)$, $(0\bar{1}10)$, and $(1\bar{1}00)$, in the ZnO NW. Thus we can easily obtain the lattice orientation of the ZnO tip via SEM imaging. Then the azimuthal angles between (0001) ZnO AFM tip and $(000\bar{1})$ ZnO substrates can be set up accurately. The azimuthal angles were adjusted by rotating the $(000\bar{1})$ ZnO substrates in the fluid cell.

Supplementary Note 4: Analyses of hydrogen bonds and surface bonds

We counted the total number of hydrogen bonds (HBs) at the separation of ~5.7 Å between the two ZnO surfaces where the normal force of the nanoparticle at 120° is close to zero for all angles. The total number of HBs (N_{HB}) includes the HBs between the two water layers, $N_{HB,W}$, and between the nanoparticle and water, $N_{HB,S}$. We used an angle-distance criterion[7] ($r_{O-O} \leq 3.3 - 0.00044\theta^2$, where θ is $\angle HO \cdots O$ in degrees). For each configuration, the geometric center (GC) of the nanoparticle in the x-y plane was calculated and only the water molecules with coordinates $|\overrightarrow{X_w} - \overrightarrow{X_{GC}}| < R_{cutoff}$ and $|\overrightarrow{Y_w} - \overrightarrow{Y_{GC}}| < R_{cutoff}$ were counted. In Supplementary Fig. 10 (blue and red lines), a cutoff radius of 10 Å that covers 92% of the surface area of the particle was used. We also counted the surface hydrogen bonds ($N_{HB,S}$, gray line of Supplementary Fig. 10) and Zn-Ow bonds (Supplementary Fig. 11) between the nanoparticle surface and the nearby water molecule where the edge atoms of the nanoparticle surface were excluded for counting. The $N_{HB,S}$ calculated by these two methods (red line for radius cutoff method and gray line for edge atom exclusion method in Supplementary Fig. 10) are very consistent. No obvious orientation dependence of the number of hydrogen bonds emerges from all three curves for number of hydrogen bonds, suggesting an entropic origin of the angular dependence. In addition, a cyclic change with a period of 60° is found in the number of Zn-O_{water} bonds in the space between the two ZnO surfaces. The bond distance of the first minimum of Zn-Ow RDF ($r_{Zn-Ow} \leq$ 3.0 Å) was used for determining Zn-Ow bonds. The total numbers of bonds were averaged over the trajectories.

Supplementary Note 5: Potential of mean force (PMF) method

A large number of PMF calculations were performed to determine the free energy of a ZnO nanoparticle interacting with a ZnO substrate at different azimuthal orientations in aqueous conditions. In the calculations, the orientation of the substrate was kept constant while the nanoparticle was initially rotated from 0° to 120° with an interval of 15°. The nanoparticle was in perfect alignment with the substrate at 0° or 120°. At each azimuthal angle, PMF calculations along the z-direction were performed and the free energy difference between the particle at height z and the particle far away from the substrate (at height z_0) was calculated by integrating the average normal force f_z of the whole nanoparticle over the distance from z_0 to z: $A(r) = \int_{\infty}^{z} \langle f_z(r) \rangle dr \approx \int_{z_0}^{z} \langle f_z(r) \rangle dr$. The reaction coordinate distance used in PMF calculations is defined as the distance between average position of the first O layer of the bottom surface and the center of mass of the top nanoparticle. But in the PMF results presented in the main text, the distance is the former distance subtracted by half of the height of the nanoparticle, close to the distance between the average position of the first O layer of the bottom surface and the average position of the bottom layer atoms of the nanoparticle. In each PMF calculation, the overall rotation about the center-of-mass (COM) of the nanoparticle about all three axes was removed. The nanoparticle was free to translate in the *x*-*y* plane.

New subroutines were implemented in DL_POLY Classic to impose the rotational and translational constraints needed to perform the PMF calculations as a function of azimuthal orientation. At the start of each simulation, the initial atomic velocities were modified, following the approach described below, to remove the initial angular momentum and COM motion of the nanoparticle. Then, at each MD step, the net torque on the nanoparticle about the three axes and the net force on the nanoparticle along the *z* axis were removed, following the approach described

below, after the force calculation and before integration of the equations of motion. In addition, because velocities are automatically scaled to the target temperature during the equilibration period, the nanoparticle's angular momentum and COM motion were also removed at each MD step during equilibration.

(1) Removal of angular momentum:

The total angular momentum about the COM of a particle can expressed as:

$$\vec{L} = \sum_i^N \vec{r_i} \times m_i \vec{V_i} = \sum_i^N m_i (\vec{r_i} \times \vec{V_i}) \quad (1)$$

where $\vec{L}$ is the total angular momentum and m_i, $\vec{r_i}$, and $\vec{V_i}$ are the mass, position relative to the COM, and linear velocity relative to the COM of atom i of a nanoparticle with N atoms, respectively.

Because of the relationship between the linear velocity and the angular velocity ($\vec{V} = \vec{\omega} \times \vec{r}$), the angular momentum can be calculated by:

$$\vec{L} = \sum_i^N m_i (\vec{r_i} \times \vec{V_i}) = \sum_i^N m_i \vec{r_i} \times (\vec{\omega_i} \times \vec{r_i}) = -\sum_i^N m_i \vec{r_i} \times (\vec{r_i} \times \vec{\omega_i}) = [I]\,\vec{\omega} \quad (2)$$

where $[I]$ is the rotational inertia.

$$[I] = \sum_i^N m_i \begin{pmatrix} y_i^2 + z_i^2 & -x_i y_i & -x_i z_i \\ -x_i y_i & x_i^2 + z_i^2 & -y_i z_i \\ -x_i z_i & -y_i z_i & x_i^2 + y_i^2 \end{pmatrix} \quad (3)$$

where x_i, y_i, and z_i are the x, y, and z components of $\vec{r_i}$.

In order to remove the net angular momentum of the nanoparticle, a uniform correction to the angular velocity of each atom should be applied as follows:

$$\Delta\vec{\omega} = [I]^{-1}\vec{L} \quad (4)$$

And thus, the correction to the linear velocity of each atom was:

$$\Delta V_i = \Delta\vec{\omega} \times \vec{r_i} \tag{5}$$

(2) Removal of torque:

The overall torque of a particle of *N* atoms can be expressed as:

$$\vec{T} = \sum_i^N \vec{r_i} \times \vec{F_i} \tag{6}$$

Similarly, the torque is related to the rotational inertia through:

$$\vec{T} = [I]\vec{\alpha} \tag{7}$$

where $\vec{\alpha}$ is the angular acceleration, which remains the same for all atoms in a rigid body.

The following shows the derivation of this equation.

$$\vec{T} = \sum_i^N \vec{r_i} \times \vec{F_i} = \sum_i^N \vec{r_i} \times (m_i \vec{a_i}) = \sum_i^N m_i\,(\vec{r_i} \times \vec{a_i}) \tag{8}$$

$$\vec{a_i} = \frac{d\vec{v_i}}{dt} = \frac{d(\vec{\omega_i} \times \vec{r_i})}{dt} = \frac{d\vec{\omega_i}}{dt} \times \vec{r_i} + \vec{\omega_i} \times \frac{d\,\vec{r_i}}{dt} = \vec{\alpha} \times \vec{r_i} + \vec{\omega} \times \vec{V_i} \tag{9}$$

By inserting the second equation into the first, the following is obtained:

$$\vec{T} = \sum_i^N m_i \left(\vec{r_i} \times (\vec{\alpha} \times \vec{r_i}) + \vec{r_i} \times (\vec{\omega} \times \vec{V_i})\right) = \sum_i^N m_i\,(\vec{r_i} \times (\vec{\alpha} \times \vec{r_i})) = [I]\vec{\alpha} \tag{10}$$

As for the correction to the angular velocities, the correction to the angular acceleration is calculated by using the inverse matrix of rotational inertia:

$$\Delta\vec{\alpha} = [I]^{-1}\vec{T} \tag{11}$$

Then, the correction to the force on each atom *i* is:

$$\Delta F_i = m_i \Delta\vec{\alpha} \times \vec{r_i} \tag{12}$$

Supplementary Note 6: Hydroxylation on polar ZnO basal surfaces

Different stabilization mechanisms were proposed for the Zn-terminated (0001) and O-terminated ($000\bar{1}$) polar surfaces. For Zn-terminated (0001) surface, a triangular surface structure was observed in scanning tunneling microscopy (STM)[8] and was found energetically favored over other surface reconstructions from DFT calculations[9]. The same DFT calculations show that when hydrogen is present, hydroxyl groups stabilize the unreconstructed Zn-terminated (0001) surface[9]. When exposed to up to 20 L water, triangular terraces were replaced by wide irregular terraces and many small pits by STM observations[10]. For O-terminated ($000\bar{1}$) surface, which is the surface that the nanoparticle approached in our study, honeycomb-like surface terminations were revealed by experiments[11] and proved to be energetically more favorable than triangular pits[12]. When exposed to water vapor, the O-terminated surface displayed a H-(1×1) pattern (i.e. 1 ML OH), revealed from He atom scattering (HAS) and low-energy electron diffraction (LEED)[13,14]. However, theoretical calculations[12,15] showed that 0.5 ML ordered H-(1 × 2) pattern is thermodynamically more favorable than H-(1×1) 1ML, which agreed with an XPS measurement[11]. The high mobility of the hydrogen atoms at the surface was proposed to explain this discrepancy[12]. As is seen from the above, no consensus on a common mechanism for stabilizing the two polar surfaces of ZnO has been reached and the mechanism depends on the surface preparation conditions. Furthermore, no report has investigated the water structure on the polar surfaces of ZnO exposed to bulk water or solution.

The well-defined H-(1×2) structure on O-terminated ($000\bar{1}$) surface (H8 in figure 6 of ref[12]) is equivalent to the isolated vacancy pattern we adopted in our calculations in terms of polarization reduction. In order to see the hydroxylation effect on the water structures on the surface, we compared the water density profiles on H-(1×2) surface the surface with isolated vacancies. The

parameters for hydroxyl-surface interactions were fitted to optimize the agreement with the geometries and adsorption energies of OH adsorption on $(10\bar{1}0)$ surface from density functional theory (DFT) calculations[4]. Both simulations were run in NVT ensemble with Nose-Hoover thermostat for 2 ns. The peak at distance equal to 0 is corresponding to the surface O atoms in both systems. The second red peak is corresponding to the surface hydroxyl oxygen. The second black peak is due to molecular water oxygen atoms sitting on the top of the vacancies (see Fig. 4(a) inset). The density of surface defects or surface molecular water in charge defect surface is about half of that of surface hydroxyl groups, that is why the second black peak is much smaller than the second red peak. In spite of a small shift of the second black peak (~ 0.5 Å) to the larger distance compared to the red second peak (Supplementary Fig. 12), the rest peak positons and heights are very similar in these two systems. In addition, both surfaces revealed the existence of at least three ordered layers of water on the $(000\bar{1})$ surface, which is consistent with the available experimental observation despite possible different surface pattern[14]. Therefore, our force field should at least qualitatively, if not semi-quantitatively capture the water structure on the polar surface, even if hydroxylated.

Supplementary References:

1 Binks, D. J. & Grimes, R. W. Incorporation of monovalent Ions in ZnO and their influence on varistor degradation. *J. Am. Ceram. Soc.* **76**, 2370-2372 (1993).

2 Berendsen, H. J. C., Grigera, J. R. & Straatsma, T. P. The missing term in effective pair potentials. *J. Phys. Chem.* **91**, 6269-6271 (1987).

3 Harris, D. J., Brodholt, J. P., Harding, J. H. & Sherman, D. M. Molecular dynamics simulation of aqueous $ZnC1_2$ solutions. *Mol. Phys.* **99**, 825-833 (2001).

4 Meyer, B., Rabaa, H. & Marx, D. Water adsorption on ZnO($10\bar{1}0$): from single molecules to partially dissociated monolayers. *Phys. Chem. Chem. Phys.* **8**, 1513-1520 (2006).

5 große Holthaus, S., Köppen, S., Frauenheim, T. & Colombi Ciacchi, L. Atomistic simulations of the ZnO($1\bar{2}10$)/water interface: a comparison between first-principles, tight-binding, and empirical methods. *J. Chem. Theory. Comput.* **8**, 4517-4526 (2012).

6 Xu, S. *et al.* Optimizing and improving the growth quality of ZnO nanowire arrays guided by statistical design of experiments. *ACS Nano* **3**, 1803-1812 (2009).

7 Wernet, P. *et al.* The structure of the first coordination shell in liquid water. *Science* **304**, 995-999 (2004).

8 Dulub, O., Diebold, U. & Kresse, G. Novel stabilization mechanism on polar surfaces: ZnO(0001)-Zn. *Phys Rev. Lett.* **90**, 016102 (2003).

9 Kresse, G., Dulub, O. & Diebold, U. Competing stabilization mechanism for the polar ZnO(0001)-Zn surface. *Phys. Rev. B* **68** (2003).

10 Önsten, A. *et al.* Water adsorption on ZnO(0001): transition from triangular surface structures to a disordered hydroxyl terminated phase. *J. Phys. Chem. C* **114**, 11157-11161 (2010).

11 Lauritsen, J. V. *et al.* Stabilization principles for polar surfaces of ZnO. *ACS nano* **5**, 5987-5994 (2011).

12 Wahl, R., Lauritsen, J. V., Besenbacher, F. & Kresse, G. Stabilization mechanism for the polar ZnO($000\bar{1}$)-O surface. *Phys. Rev. B* **87** (2013).

13 Kunat, M., Girol, S. G., Burghaus, U. & Wöll, C. The interaction of water with the oxygen-terminated, polar surface of ZnO. *J. Phys. Chem. B* **107**, 14350-14356 (2003).

14 Schiek, M., Al-Shamery, K., Kunat, M., Traeger, F. & Wöll, C. Water adsorption on the hydroxylated H-(1× 1) O-ZnO ($000\bar{1}$) surface. *Phys. Chem. Chem. Phys.* **8**, 1505-1512 (2006).

15 Meyer, B. First-principles study of the polar O-terminated ZnO surface in thermodynamic equilibrium with oxygen and hydrogen. *Phys. Rev. B* **69**, 45416 (2004).
