# Supplementary material for: Direction-specific interaction forces underlying zinc oxide crystal growth by oriented attachment
Source: Nat Commun. 2017 Oct 10;8:835. doi: 10.1038/s41467-017-00844-6 (PMC5635138; doi:10.1038/s41467-017-00844-6)
Supplement: Supplementary file 2 — Description of Additional Supplementary Files [file 41467_2017_844_MOESM2_ESM.pdf]

Description of Additional Supplementary Files

File Name: Supplementary Movie 1
Description: Dynamic force measurement.

File Name: Supplementary Movie 2
Description: In-situ AFM movie for ZnO($000\overline{1}$) substrate in 0.2 mM $Zn(NO_3)_2$ solution.

File Name: Supplementary Movie 3
Description: MD trajectories of ZnO nanoparticle interacting with ZnO 2-D slab in aqueoues environment .
